# Morphological and Radiologic Features of the Skull of a Juvenile Green Turtle (*Chelonia mydas*, Linnaeus, 1758) from Saint Kitts and Nevis, West Indies

**DOI:** 10.3390/ani16060990

**Published:** 2026-03-22

**Authors:** Cristian Olimpiu Martonos, Cristian Constantin Dezdrobitu, Gilda Rawlins, Calin Lațiu, Alexandru Ion Gudea

**Affiliations:** 1 Department of Biomedical Sciences, Ross University School of Veterinary Medicine, Basseterre P.O. Box 334, Saint Kitts and Nevis; cmartonos@rossvet.edu.kn (C.O.M.); cdezdrobitu@rossvet.edu.kn (C.C.D.); 2 Department of Clinical Sciences, Ross University School of Veterinary Medicine, Basseterre P.O. Box 334, Saint Kitts and Nevis; grawlins@rossvet.edu.kn; 3 Faculty of Animal Husbandry and Biotechnologies, University of Agricultural Sciences and Veterinary, Medicine Cluj-Napoca, Romania, Calea Mănăstur 3-5, 400372 Cluj-Napoca, Romania; calin.latiu@usamvcluj.ro; 4 Department of Anatomy, Faculty of Veterinary Medicine, University of Agricultural Sciences and Veterinary, Medicine Cluj-Napoca, Romania, Calea Mănăstur 3-5, 400372 Cluj-Napoca, Romania

**Keywords:** green turtle, *Chelonia mydas*, anatomy, cranium, diagnostic imaging, West Indies

## Abstract

The skull of a juvenile *Chelonia mydas* from the beaches of Saint Kitts and Nevis is described using high-resolution photographs and multi-angle radiographs to document the main cranial structures. Standard veterinary anatomical terminology was used, and osteometric measurements were obtained and compared with published data on adult individuals. The investigated juvenile green turtle skull comprises three main regions: the neurocranium, splanchnocranium, and mandible. Noteworthy features include a paired premaxilla at the snout tip, a prominent L-shaped maxilla forming much of the upper jaw, and a robust parietal bone at the skull’s apex. Additional observations include the presence of a lacrimal foramen and a distinct crest on the exoccipital bone. As the study is constrained by reliance on a single juvenile specimen, the findings should be interpreted as preliminary rather than as strong indicators of the population. Morphometric data suggest the juvenile skull reaches about 40% of adult size. Measurements show somewhat greater growth in the posterior region than in the anterior region, which might imply later development or changes in the feeding structures of the skull. Overall cranial proportions remain consistent during growth. Because the metrical study relies on a single juvenile specimen, findings should be interpreted as preliminary rather than as strong indicators of the population. These results, combined, may serve as an entry point for future taxonomic, evolutionary, and clinical research, as comprehensive anatomical knowledge of both local and migratory species, such as *Chelonia mydas*, is essential for conservation initiatives, veterinary diagnostics, and the study of adaptive morphological changes in isolated island ecosystems.

## Simple Summary

A comprehensive anatomical and radiological analysis of the skull of a juvenile green sea turtle (*Chelonia mydas*) from Saint Kitts and Nevis is presented. Three primary cranial regions are identified: the neurocranium, splanchnocranium, and mandible. Notable features include the lacrimal foramen and a U-shaped jaw, which are indicative of herbivorous adaptation. Morphometric data indicate that the juvenile specimen has likely reached approximately 40% of adult size, with growth patterns suggesting that the posterior braincase may develop ahead of the anterior part of the skull region. However, further evidence would help confirm this sequence.

## 1. Introduction

Comprehensive anatomical documentation of local and migratory species within insular ecosystems is essential for understanding evolutionary adaptations and for supporting conservation efforts and targeted veterinary care. Detailed morphological references for local species [1,2,3,4,5,6,7,8,9] provide a foundation for comparative anatomy, functional studies, and the development of evidence-based health management strategies.

Historically, the Caribbean Islands and their large green turtle population played a crucial role for the crews of ships travelling to and from Africa, as their eggs and meat were an important source of protein [10,11,12,13].

Saint Kitts is an oceanic island of 176 km^2^, part of the Saint Kitts and Nevis Federation, together with its twin-island, Nevis [13]. According to [14], oceanic islands are very important for their high biodiversity, yet they host approximately 33% of the world’s endangered vertebrates.

Taxonomically, sea turtles belong to two clades, *Dermochelyidae* and *Cheloniidae* [15], and are divided into 7 species [9,16]. The first clade includes only one representative, *Dermochelys coriacea*, while the second clade includes the remaining six species: *Caretta caretta*, *Chelonia mydas*, *Eretmochelys imbricata*, *Lepidochelys kempii*, *Lepidochelys olivacea*, *Natator depressus* [9,15].

Among the named species, the green turtle (*Chelonia mydas*) is the largest representative of the *Cheloniidae* clade and the second-largest, after *Dermochelys coriacea*, with literature reporting adult specimens weighing 150 kg and with a carapace length of 95 to 120 cm [12]. Geographically, green turtles are found worldwide, inhabiting tropical and subtropical coastal waters. A pronounced migratory behaviour has been reported in *Chelonia mydas*. According to [17], for these turtles, nesting and feeding areas are usually different, which explains the reported seasonal migrations [17,18,19].

Throughout their lives, their nutritional behaviour shows different characteristics. Initially, juveniles have an omnivorous diet based on pleustonic and planktonic material, and occasionally on small animals adhering to the seagrass [20,21]. Important changes have been reported in adults, who are predominantly herbivores [9,19,21]. This transition to vegetarian alimentation occurs when the juvenile carapace reaches a length of 25–35 cm [20]. The morphological changes that occur at the level of the skull from the juvenile to the adult have been indicated as a possible cause of the switch from an omnivore/carnivore diet to an herbivore diet [19].

Previous morphological studies of sea turtles were conducted to pinpoint macroscopic differences between species [22,23], to identify regional variability [24,25], to examine swimming performance in hatchlings [26], or to identify skull differences between juveniles and adults. Valuable morphological data have been reported previously on the heads and crania of different sea turtles [9,16,19,23,27,28,29,30].

Despite the limitations of a single juvenile specimen, this study also adds specific knowledge, given the limited osteological data available, and offers preliminary morphological and imaging data, along with initial morphometric data on the bony components of the splanchnocranium and neurocranium in *Chelonia mydas* from the beaches of Saint Kitts and Nevis. These findings may serve as a basic reference to help familiarise examiners with the species’ general cephalic features.

No generative artificial intelligence (GenAi) has been used in this paper.

## 2. Materials and Methods

### 2.1. Morphological Investigation

The present study was conducted in the anatomy laboratory at Ross University School of Veterinary Medicine in Basseterre, Saint Kitts and Nevis. It used one (1) juvenile skeleton of *Chelonia mydas*, Linnaeus 1758, part of the St. Kitts Sea Turtle Monitoring Network collection. The individual’s age was unknown, but was subjectively evaluated based on external features (beak shape, carcass size, and shape) as the specimen was found in a state of putrefaction on a public beach in St Kitts. The carcass came into the possession of a member of the St. Kitts Sea Turtle Monitoring Network (SKSTMN), who lended the prepared specimen to the RUSVM Laboratory in 2010 for study purposes, after the usual preparation for bone specimens. To the present day, the specimen is part of the SKSTMN collection with no inventory or collection data.

To obtain relevant information, photographs have been taken in sequence. Each photo had added a measurement scale. For high-quality images, the Canon EOS 90D (Canon, Melville, NY, USA) was used. Adobe Photoshop^®^ 21 (Adobe Itl, Atlanta, GA, USA) has been used to make minor improvements and enhancements to the photos (black background).

We carefully assessed morphological features and reported the surfaces and margins of each bone. The terminology used to name the anatomical structures is in accord with *Nomina Anatomica Veterinaria* [31] and *Nomina Anatomica Avium* [32].

Radiographic investigations were performed using the Vet Ray Technology Standard Vet device (Niles, IL, USA) at 85 kVp and 3.2 mAs at the RUSVM Veterinary Clinic, with proper scale markers and standard exposure, ray alignment, and specimens placed in standard positions.

### 2.2. Osteometrical Investigation

The metrical investigation of the studied specimen is important as the number of measurements available for this species is very limited. A comparative osteometrical data set may be relevant for studies examining different taxonomic relationships among groups and can provide significant data to the existing published dataset.

The possible measurements (Table 1) performed on the available specimens are listed below, along with the widely used nomenclature in specialised literature [22,33,34].

To reduce bias, a series of 3 seriate measurements was taken for each of the listed sets at different time intervals. Image analysis was performed in ImageJ 1.530 [35] with the embedded ruler tool.

Due to the very limited metrical data (only 1 juvenile individual) in our sample, a simple comparison with the existing literature was performed, aiming to provide only a basic metrical integration of these measurements.

## 3. Results

### 3.1. Morphological Data

Anatomically, three main divisions of the skull are listed: the splanchnocranium, the neurocranium, and the mandible.

The elements that form the neurocranium are part of the inner brain case, while the components of the splanchnocranium form the external superstructure of the green turtle’s skull. Their surfaces provide muscle attachments for a significant number of muscles.

**Figure 1:**
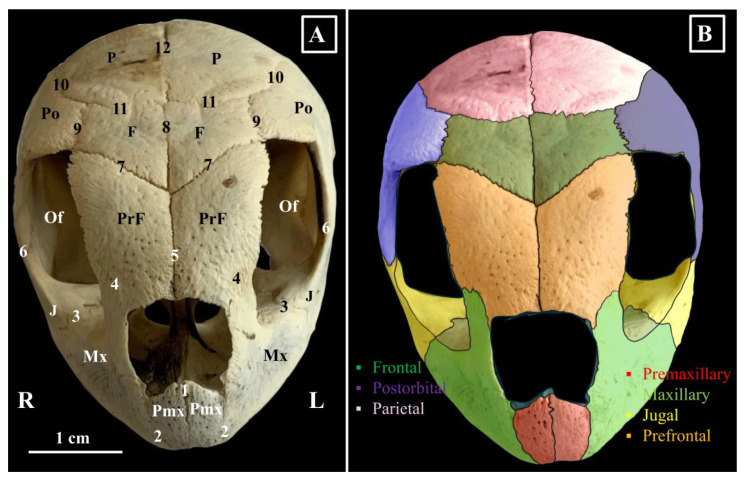
Green turtle skull-morphological characteristics, rostral view. (**A**)—bones and sutures; (**B**)—Colourisation of the dorsal part of the skull in the Green turtle. (**A**). F—Os frontale; J—Os jugale; Mx—Os maxillare; Of—fossa orbitalis; Pmx—Os premaxilare; PrF—Os prefrontale; Po—Os postorbitale; P—Os parietale; 1. Sutura interpremaxillaris; 2. Sutura maxillo-premaxilaris; 3. Sutura maxillo-jugalis; 4. Sutura maxillo-prefrontalis; 5. Sutura interprefrontalis; 6. Sutura jugulo-postorbitalis; 7. Sutura fronto-prefrontalis; 8. Sutura interfrontalis; 9. Sutura fronto-postorbitalis; 10. Sutura parieto-postorbitalis; 11. Sutura parieto-frontalis; 12. Sutura interparietalis.

**Figure 2:**
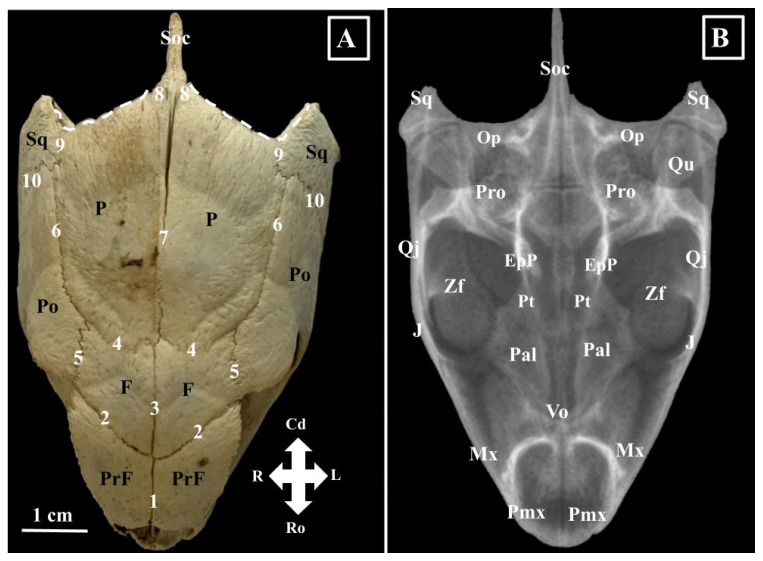
(**A**)—Green turtle skull-morphological characteristics, dorsal view (**B**)—Skull radiograph dorso-ventral projection. (**A**). 1. Sutura interprefrontalis; 2. Sutura fronto-prefrontalis; 3. Sutura interfrontalis; 4. Sutura parieto-frontalis; 5. Sutura fronto-postorbitais; 6. Sutura parieto-postorbitalis; 7. Sutura interparietalis; 8. Parieto-supraoccipital suture; quadrat; 9. Parieto-squamosal suture; 10. Sutura squamosal-postorbitalis; F—Os frontalis, PrF—Os prefrontalis; Po—Os postorbitalis; P—Os parietalis; Soc—Os supraoccipitalis; Sq—Os squamosum; Ro—rostral; Cd—caudal; White dotted lines—parietal notches. (**B**). EpP—Os epipterygoideum; J—Os jugale; Mx—Os maxillaris; Op—Os opisthoticum; Pal—Os palatinum; Pt—Os pterygoideum; Pmx—Os premaxilaris; Pro—Os prooticum; Qj—Os quadratojugale; Qu—Os quadratum; Soc—Os supraoccipitalis; Sq—Os squamosum; Vo—Os vomer; Zf—Fossa zygomatica.

**Figure 3:**
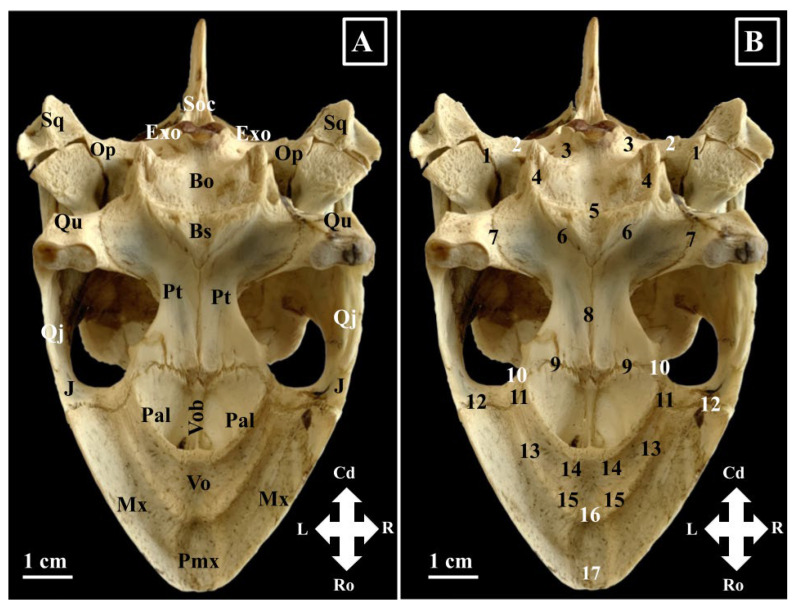
Green turtle skull-morphological characteristics, ventral view (**A**)—bones; (**B**)—sutures. (**A**). Bo—Os basioccipitalis; Bs—Os basisphenoidalis; Exo—Os exoccipitalis; J—Os Jugalis; Mx—Os maxillaris; Pal—Os palatinum; Pt—Os pterygoideum; Pmx—Os Premaxillararis; Qu—Os quadratum; Qj—Os quadratojugalis; Op—Os opisthoticum; Sq—Os squamosum; Soc—Os supraoccipitale; Vo—Os vomer; Vob—corpus vomeris. (**B**). 1. Sutura opistothotico-quadrata; 2. Sutura opisthotico-exoccipital; 3. Sutura exoccipital-bisoccipitalis; 4. Sutura pterygo-occipitalis; 5. Sutura occipito-sphenoidalis; 6. Sutura pterygo-sphenoidalis; 7. Sutura pterygo-quadrata; 8. Sutura interpterygoidea; 9. Sutura pterygo-palatina; 10. Sutura pterygo-jugalis; 11. Sutura jugo—palatina; 12. Sutura jugo-maxilaris; 13. Palatomaxillar sutures; 14. Sutura vomero-palatina; 15. Sutura vomero-maxillaris; 16. Sutura vomero—premaxilaris; 17. Sutura interpremaxilaris.

The premaxilla (*Os premaxillare*) (Figure 1, Figure 2 and Figure 3) is a paired bone. It has two surfaces and five margins: a rostral surface (*Facies rostralis*) and a caudal one (*Facies caudalis*), a maxillary margin (*Margo maxillaris*), a palatinal margin (*Margo palatinus*), a nasal margin (*Margo nasalis*), and a sagittal margin (*Margo sagitallis*) (Figure 1).

Macroscopically, this bone has a checkmark aspect and displays a rostral rectangular plate and a caudal process—the palatine process (*Processus palatinus*) of the premaxillary bone. The rostral surface is smooth and faces rostrally. The maxillary margin of the premaxilla articulates caudo-laterally with the rostral segment of the maxillary bone. The sagittal margin articulates with the symmetrical premaxillary bone. The palatine process articulates caudally with the rostral segment of the vomer bone (*Vomer*), laterally with the internal surface of the maxillary bone (*Os maxillare*) and in a sagittal plane with the symmetrical homologue. The caudal processes of the left and right premaxillary bones form the rostral segment of the hard palate. The dorsal aspect of these pieces can be considered the nasal surface of the premaxilla, while the ventral aspect forms the buccal surface of the premaxilla. The dorsal margin of the premaxillary rostral plate, together with the rostro-dorsal segment of the maxillary bone and the rostral end of the prefrontal bone, provides the anatomic base for the external nares. In the median plane, the dorsal border of the rectangular plates continues dorsally with a sharp bony process, which forms the external nasal orifice in two halves.

**Figure 4:**
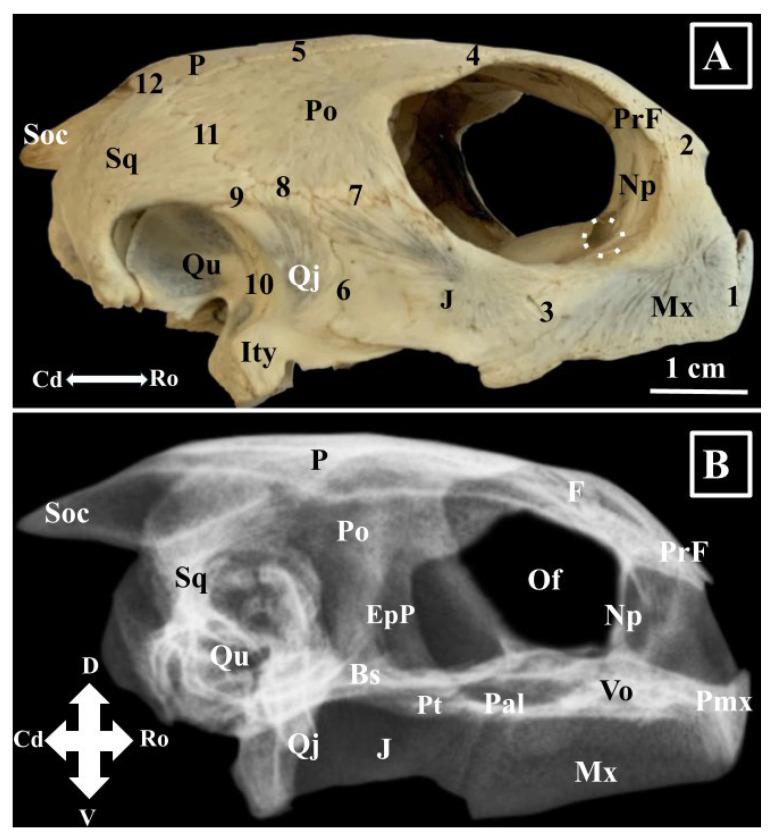
Green turtle skull-morphological characteristics, right lateral view (**A**)—bones; (**B**)—Skull radiograph—latero-lateral projection. J—Os jugale; Mx—Os maxilaris; Np—Os prefrontalis processus nasalis; P—Os parietale; Po—Os postorbitalis; PrF—Os prefrontalis; Qj—Os quadratojugalis; Qu—Os quadratum; Soc—Os supraoccipitale; Sq—Os squamosale; Ro—rostral; Cd—caudal; White dotted circle-foramen lacrimalis. 1. Sutura maxillo-premaxillaris; 2. Sutura maxillo-prefrontalis; 3. Sutura maxillo-jugalis; 4. Sutura fronto-postorbitalis; 5. Sutura parieto-postorbitalis; 6. Sutura jugulo-quadrata; 7. Sutura jugo-postorbitalis; 8. Sutura quadratojugal-postorbitalis; 9. Sutura squamosoquadratojugalis; 10. Sutura quadratoquadratojugalis; 11. Sutura squamoso-postorbitalis; 12. Sutura parieto-squamosalis.

The maxillary bone (*Os maxillare*) (Figure 1, Figure 2, Figure 3 and Figure 4) is one of the largest elements of the splanchnocranium. Due to its position, this bone presents four surfaces: a lateral surface (*Facies facialis*), an orbital surface (*Facies orbitalis*), a nasal surface (*Facies nasalis*), and a buccal surface (*Facies buccalis*). With an L-shaped and a smooth aspect, the lateral surface of the maxillary bone is the larger one. Rostrally, it joins the premaxillary bone (*Sutura maxillo-premaxillaris*), caudally the jugal bone (*Sutura maxillo-jugale*), ventromedially the palatine (*Sutura palatomaxillaris*) and vomer (*Sutura vomeromaxillaris*) bones.

Dorso-rostrally, the lateral surface of the maxillary bone bends and forms the rostro-ventral segment of the orbital cavity (*Cavitas orbitalis*). The orbital surface is small compared with the lateral surface and forms a part of the ventro-rostral floor of the orbital cavity. This surface merges caudally with the orbital segment of the jugular bone (*Suture maxillo-jugale*), mediodorsally with the orbital surface of the prefrontal bone (*Sutura maxillo-prefrontale*), and mediodorsally with the orbital surface of the palatine bone (*Sutura palatomaxillaris*). Between the lateral and orbital surfaces, the maxillary segment of the orbital crest (*Margo orbitalis*) can be identified. The nasal surface is also small and provides the anatomic base of the latero-ventral walls of the nasal cavity (*Cavum nasi*). The buccal surface forms the latero-dorsal bony walls of the buccal cavity (*Cavum bucalis*). Medially, we observed that a palatine process had been sent. On their buccal surfaces, the left and right palatine processes bear two bony crests that are parallel with the ventral margins of the maxillary bones.

The palatine processes of the maxillary bone, together with the palatine bones, the vomer, and the palatine processes of the premaxillary bones, form the hard palate, the boundary between the buccal and nasal cavities. The ventral edge of the premaxilla rostrally, combined with the ventral edge of the maxilla laterally, forms the hard palate and gives it a U-shaped appearance.

The prefrontal bone (*Os prefrontalis*) (Figure 1, Figure 2 and Figure 4) is a rectangular bony piece that forms the entire roof of the nasal cavity and the rostro-dorsal segment of the orbital cavity. The external surface is convex, and the internal surface is concave. This last surface projects a ventral bony process that forms the caudal wall of the nasal cavity and the rostral wall of the orbital cavity. This last structure can be named as the nasal process of the prefrontal bone (*Os prefrontalis processus nasalis*). This process reaches the dorsal surface of the hard palate. Between its medial margins, the ethmoidal fissure (*Fissura ethnoidalis*) can be observed. This space has a tear-like aspect and a dorsal disposition. The lacrimal foramen perforates the distal end of the nasal process (*Foramen lacrimale*) (Figure 3), a structure that allows communication between the orbital and nasal cavities.

The rostral margin of the prefrontal bone forms the dorsal border of the external nares, and its rostro-lateral segment meets with the maxillary bone. The lateral edge forms the dorso-lateral margin of the orbital cavity, and the other two margins articulate with different bones. The medial margin has a sagittal disposition and meets with the medial margin of the opposite prefrontal bone (*Sutura interprefrontalis*). The caudal margin has an oblique disposition and joins with the rostral margin of the frontal bone (*Sutura fronto-prefrontalis*). Both right and left fronto-prefrontal sutures form a V-shaped aspect with a caudal opening.

The frontal (*Os frontale*) (Figure 1 and Figure 2) bone is a rectangular bone with two surfaces (an internal and an external surface) and four margins (sagittal, rostral, caudal, and lateral).

The external surface (*Facies externa*) is smooth and slightly convex, and the internal surface (*Facies interna*) is concave and bears the orbitosphenoidal crest (*Crista orbitosphenoidalis*). Rostrally, the orbitosphenoidal crest continues with the border of the ethmoidal fissure and caudally with the ventral alisphenoid process of the parietal bone. This structure divides the ventral surface into two unequal segments. The lateral segment is larger and forms the dorso-caudal segment of the roof of the orbital cavity, while the medial segment is smaller and forms a part of the roof of the cranial cavity. This segment, in live specimens, accommodates the nervous bandelets which continue caudally to the olfactory bulbs (*Bulbus olfactorius*), the olfactory peduncles (*Pedunculus olfactorius*).

The rostral margin can also be named the prefrontal margin (*Margo prefrontalis*). It has an oblique caudolateral direction, and its latero-caudal point enters between the prefrontal and postorbital bones (*Os postorbitale*). This border allows articulation with the prefrontal bone. The lateral border runs in a caudal direction and is parallel with the dorsal segment of the orbital and meets with the rostro-dorsal process of the postorbital bone (*Sutura fronto-postorbilalis*). The caudal margin, the parietal one (*Margo parietalis*), joins with the rostral margin of the parietal bone (*Sutura fronto-parietalis*), and the sagittal margin allows contact with the opposite frontal bone (*Sutura interfrontalis*).

The postorbital bone (*Os postorbitale*) (Figure 1, Figure 2 and Figure 4) articulates with the frontal bone (*Sutura fronto-postorbilalis*) rostro-medially, with the parietal bone (*Sutura parieto-postorbitalis*) dorso-medially, with the squamosal (*Sutura squamoso-postorbitalis*) bone caudally, the quadratojugal bone ventro-caudo-laterally, and the jugal bone (*Sutura jugo-postorbitalis*) ventro-rostro-laterally.

Its rostral margin forms the caudo-dorsal segment of the orbital margin. The external surface is smooth and bends dorso-laterally. The internal surface presents two distinctive areas. A small lacrimal area, which has a dorso-rostral position accommodating the lacrimal gland and a slightly larger temporal area with a dorso-lateral position that is part of the temporal fossa (*Fossa temporalis*).

The jugal bone (*Os jugale*) (Figure 1, Figure 2, Figure 3 and Figure 4) forms the caudal segment of the ventral orbital floor and the ventro-caudal segment of the orbital margin. Its margins allow connections with the postorbital bone dorsally (*Sutura jugo-postorbitalis*), the quadratojugal bone caudally, the maxillary bone rostrally, the palatine bone rostro-medially (*Sutura palato-jugalis*), and with the pterygoid bone medially (*Sutura pterygo-jugalis*). The external surface is smooth, but the medial surface features a bony projection, the pterygoid process (*Processus pterygoideus*), which extends medially. Rostrally, this process connects with the orbital segment of the maxillary bone.

The quadratojugal bone (*Os quadratojugale*) (Figure 2, Figure 3 and Figure 4) is a bony lamina, surrounded dorsally by the squamosal and postorbital bones, rostrally by the jugal bone, and caudally by the quadrate bone. Its caudal margin, the tympanic margin (*Margo tympanicum*), has a caudal concavity and provides the rostral limit of the *cavum tympani*. The ventro-caudal segment of the quadratojugal bone covers the lateral aspect of the ventral segment of the quadrate bone and forms the infratympanic process (*Processus infratympanicum*).

**Figure 5:**
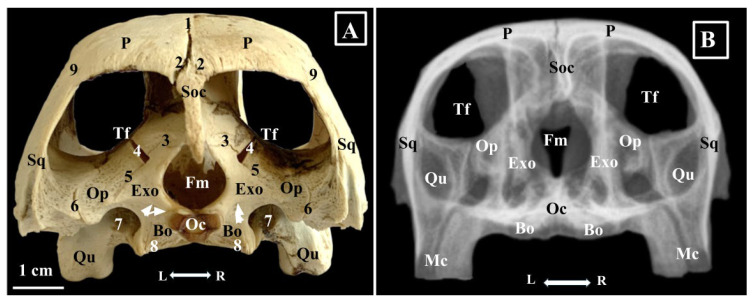
(**A**)—Green turtle skull-morphological characteristics, caudal view. (**B**)—Skull radiograph rostro-caudal projection. Bo—Os basioccipitalis; Exo—Ossa exoccipitale; Fm—Foramen magnum; Qu—Os quadratum; Oc—Condylus occipitalis; Op—Os opisthoticum; Sq—Os squamosum; P—Os parietale; Soc—Os supraoccipitale; Tf—Fossa temporalis; White arrows—Canalis n. hypoglosi. 1. Sutura interparietalis; 2. Sutura parieto-supraoccipitalis; 3. Sutura exocipital-supraoccipitalis; 4. Sutura Opisthotico-supraoccipitalIS; 5. Sutura opisthotico-exoccipitalis; 6. Sutura opisthotico-squamosalis; 7. Sutura opisthotico-exoccipitalis; 8. Sutura exoccipital-bisoccipitalis; 9. Sutura parieto-squamosalis. L—left; R—Right.

The quadrate bone (*Os quadratum*) (Figure 2, Figure 4 and Figure 5) has a caudal location with an excavated external surface (which is part of the *cavum tympanicum*). Due to its position, the squamosal (*Sutura squamosoquadrate*) covers it dorso-caudally, the opisthotic bone meets it medio-caudally, and the prootic bone meets it medio-rostrally. Rostrally, it joins the quadratojugal bone and forms the caudal wall of the zygomatic fossa. The ventral projection bears a flat articular process (*Processus mandibularis*), part of the craniomandibular joint.

The squamosal bone (*Os squamosum*) (Figure 2, Figure 3, Figure 4 and Figure 5) builds the caudo-lateral wall of the temporal fossa (*Fossa temporalis*). Rostrally, it articulates with the parietal, postorbital, and quadratojugal bones. The joint areas with the parietal and quadratojugal are very short, compared to the squamoso-postorbital joint (*Sutura squamoso-postorbitalis*), which is very long. The quadrate, opisthotic, and prootic bones come in contact with the medial surface of the squamosal bone. Ventrally, its caudal margin allows identification of two bony crests. The medial one (*Crista medialis*) is shorter and more evident compared with the lateral one (*Crista lateralis*), which is longer. The groove located between these two crests accommodates the lateral head of the squamoso-maxillaris muscle (*Sulcus squamoso-maxilaris*). The ventral margin is curved and forms the dorso-posterior limit of the *cavum tympanicum*.

The parietal bone (*Os parietale*) (Figure 1, Figure 2, Figure 4 and Figure 5) is the largest bone of the skull in Green turtles and, due to its position, forms the dorsal wall of the cranial cavity, the dorsal wall of the temporal fossa, and the dorso-caudal wall of the orbital cavity. Its ventral surface sends a bony lamina, the alisphenoid plate, or the vertical ventral processes of the parietal bone (*Ala major ossis sphenoidalis*). This bony process divides the internal surface of the parietal bone into two unequal areas. The medial one is the cerebral segment, and together with the opposite forms the roof of the neurocranium. The lateral one is larger and forms the roof of the temporal fossa and the caudo-dorsal segment of the orbital fossa. The caudal margins of the temporal bone showed a reduced parietal notch (*Incisura parietalis*). Rostrally, the temporal bone articulates with the caudal margin of the frontal bone (*Sutura fronto-parietalis*), rostro-laterally and laterally with the postorbital bone (*Sutura parieto-postorbitale*), latero-caudally with the squamosal bone (*Sutura squamoso-parietalis*), caudo-sagittally with the supraoccipital bone (*Sutura squamoso-supraoccipitalis*) and with the basisphenoid bone ventrally (*Sutura squamoso-basisphenoidalis*).

The palatine bone (*Os palatinum*) (Figure 2 and Figure 3) is a paired bone that presents a buccal (*Processus buccalis*) and an orbital (*Processus orbitalis*) segment. The buccal segment is smaller and lies horizontally; together with the vomer and the opposite palatine process, it forms the hard palate. It has a dorso-vertical position and forms the caudo-dorso-lateral walls of the nasal cavity and the rostro-ventro-medial segment of the orbit. The rostral border of this segment forms the ventro-caudal border of the lacrimal foramen. The palatine bone meets rostrally with the vomer (*Sutura vomero-palatina rostralis*), rostro-laterally with the maxillary bone (*Sutura maxillo-palatina*), caudo-laterally with the pterygoid process of the jugal bone (*Sutura jugo-palatina*), with the vomerian body (*Sutura vomero-palatina dorsalis*) dorsally, with the pterygoid bone (*Sutura pterygo-palatina*) caudally and with the nasal process of the prefrontal bone (*Sutura prefronto-palatina*) rostro-dorsally.

The vomer bone (*Os vomer*) (Figure 2 and Figure 3) has two parts. The dorsal part occupies the dorso-sagittal plane of the roof of the nasal cavity and the ventro-sagittal plane of the floor of the orbital cavity and is considered the body of the vomer (*Corpus vomeris*). Being located between vertical blades of the palatine bones and on top of the palatine processes of the premaxilla bones, the dorsal surface of the vomer body allows identification of a bony groove, the ethmoidal groove (*Sulcus ethmoidalis*). The ventral part is horizontally oriented and rhomboidal. Its ventral surface has a central excavation and forms the largest part of the palate. Its dorsal surface is convex and bears a bony crest that connects it with the body of the vomer. The vomerian body articulates with the pterygoid bone caudally (*Sutura vomero-pterygoidea*), with the vertical segment of the palatine bone dorso-laterally (*Sutura vomero-palatina dorsalis*), with the horizontal segment of the palatine bone ventro-laterally (*Sutura vomero-palatina rostralis*), with the maxillary bone laterally (*Sutura vomero-maxilaris*), and with the palatine processes of the premaxillary bone rostrally (*Sutura vomero-premaxillaris*). The bony crest, which connects the body with the blade, forms the median septum between the right and left internal nares.

The pterygoid bone (*Os pterygoideum*) (Figure 2 and Figure 3) is a paired bone that forms the anatomic base of the roof of the pharyngeal cavity. Articulated on the sagittal midline, the ventral aspect of the pterygoid bones allows us to observe an hourglass-shaped aspect, with a narrow mid-portion and wider extremities. The lateral margins of the pterygoid bones form the medial border of the zygomatic fossa (*Fossa zygomatica*). Rostrally, this bone joins the caudal margin of the palatine bone (*Sutura pterygo-palatina*) and rostro-laterally articulates with the pterygoidean process of the jugal bone (*Sutura pterygo-jugalis*). Caudo-medially, it articulates with the basisphenoid bone (*Sutura pterygosphenoidalis*) and caudo-laterally with the inner surface of the quadrate bone. On its latero-dorsal surface, the pterygoid bone meets the epipterygoid bone (*Ossa epipterygoideum*) via two epipterygoid processes (*Processi epipterygoideus*).

The epipterygoid bone (*Os epipterygoideum*) (Figure 2) is a triangular bony lamina with a wide base oriented dorsally. It meets the alisphenoid plate or the vertical ventral processes of the parietal bone (*Sutura parietoepipterygoida*) and ventrally articulates with the epipterygoid process of the pterygoid bone (*Sutura pterygo-epipterygoida*).

The basioccipital bone (*Os basioccipitale*) (Figure 3 and Figure 5) is an unpaired bone of the neurocranium that forms the caudal part of the floor of the cranial cavity and the ventral one-third of the occipital condyle (*Condylus occipitalis*). Due to its position, a small ventral segment of the occipital orifice (*Foramen magnum*) is also built by it. Its latero-caudal margins articulate with the exooccipital bones, and rostrally, it articulates with the caudal margin of the basisphenoid bone (*Os basisphenoidale*).

The supraoccipital bone (*Os supraoccipitale*) (Figure 2, Figure 3, Figure 4 and Figure 5) is an unpaired bone that forms the caudo-dorsal segment of the roof of the cranial cavity and is listed as an element of the braincase. Caudally in *Chelonia mydas*, it continues with a well-developed supraoccipital crest (*Crista supraoccipitale*). The external surface of the supraoccipital bone is small and covered partially by the caudal portion of the parietal bone. The inner surface is concave and forms the dorsal part of the occipital foramen and the dorso-lateral part of the caudal extremity of the cranial cavity. Inside the cranial cavity, the ventral surface of the supraoccipital bone sends two ventral processes. This bone is connected dorsally to the parietal bones, ventrolaterally to the opisthotic and prootic bones. As part of the occipital foramen (*Foramen magnum*), it articulates with the exoccipital bones (*Ossa exoccipitalia*). Rostrally, its ventral processes meet the vertical ventral plates of the parietal bone. The supraoccipital bone together with the right and left exoccipital bones and the basioccipital bone (*Os basioccipitale*) form the occipital bone (*Os occipitale*).

The exoccipital paired bones (*Ossa exoccipitale*) (Figure 3 and Figure 5) form the caudal wall of the cranial cavity and the latero-ventral segments of the occipital foramen and the lateral two-thirds of the occipital condyle. Laterally to the occipital condyle, a small excavated area displays marks of the external opening of the hypoglossal canals (*Canalis n. hypoglossi*).

The basisphenoid bone (*Os basisphenoidale*) (Figure 3) fills the space between the basioccipital bone caudally and the pterygoid bones rostro-laterally and forms the rostral segment of the floor of the cranial cavity. The main part of the bone is the body. It continues rostrally with a rostral process (*Processus rostrale*) which runs on the dorsal surface of the interpterygoidean suture, and caudally with two basipterygoid processes (*Processus basipterygoideum dextrum*/*Processus basipterygoideum sinister*) which run latero-caudally.

The opisthotic bone (*Os opisthoticum*) (Figure 2, Figure 3 and Figure 5) fills the space between the exoccipital and supraoccipital bones medially and the squamosal and quadrate bones laterally. Ventrally, together with the quadrate bone dorso-laterally and the exoccipital bone dorso-medially, the opisthotic bone forms the roof of the acustico-jugal cavity.

The prootic bone (*Os prooticum*) (Figure 2) forms the rostro-medial segment of the temporal fossa and the caudo-medial border of the zygomatic fossa. Its inner surface, together with the inner surface of the opisthotic and supraoccipital bones, forms the lateral walls of the cranial cavity. It articulates with the opisthotic bone caudally, with the quadrate and quadratojugal bones laterally, and with the pterygoid and epipterygoid bones rostrally. The junction with the quadrate bone bears the foramen stapedio-temporalis for the stapedial artery.

**Figure 6:**
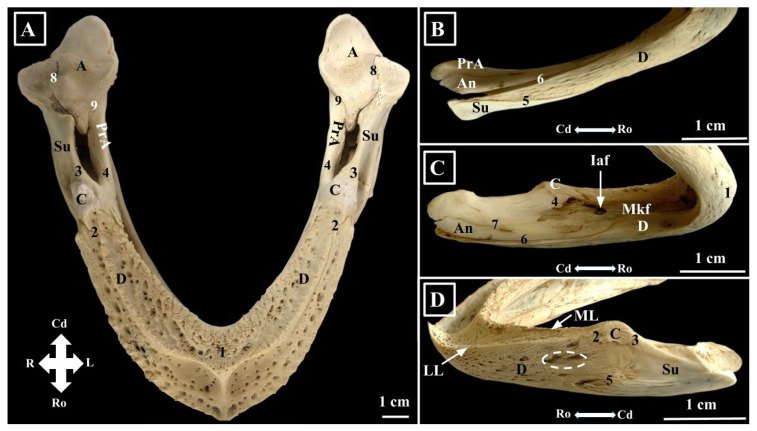
Green turtle mandible-morphological characteristics, (**A**)—Dorsal view; (**B**)—Ventral view; (**C**)—Medial view; (**D**)—Lateral view. A—Os articulare; An—Os angulare; C—Os cornoideum; D—Os dentale; Iaf—Foramne mandibulae; LL—Linea labialis/lateralis; ML—Linea lingualis; PrA—Os prearticulare; Su—Os surangulare; White dotted circle—Foramen dentofaciale majus. 1. Symphysis mandibulae; 2. Sutura corono-dentalis; 3. Sutura corono-surangularis; 4. Sutura corono-prearticularis; 5. Sutura dento-surangularis; 6. Sutura dento-angularis; 7. Sutura angulo-prearticularis; 8. Sutura articulo-surangularis; 9. Sutura articulo-prearticularis; Mkf—Fossa meckelii; Cd—Caudal; L—left; R—Right; Ro—rostral.

The mandible (*Mandibula*) (Figure 6) is the mobile component of the skull and caudally articulates with the mandibular condyle of the quadrate bone (*Articulatio quadrato-mandibulare*). It is a composite bony structure that allows identification of six paired bones: dentary (*Os dentale*), coronoid (*Os coronoideum*), angular (*Os angulare*), surangular (*Os surangulare*), prearticular (*Os prearticulare*), and articulare (*Os articulare*).

The rostral segment of the mandible (*Rostrum mandibulae*) is formed by the junction of the right and left dentary bones (*Os dentale dextrum et Os dentale sinistrum*) (Figure 6) at the level of the mandibular symphysis (*Symphysis mandibulae.* The ventral surface (*Facies ventralis*) is rounded and caudally continues with a sharp postero-ventral process, the angular process (*Processus angularis*), which meets the surangular bone laterally, the angular bone medially and the articular bone caudo-dorsally. The dorsal surface (*Facies dorsalis*), also known as the alveolar surface (*Facies alveolaris*), has a rectangular rostral aspect and bears a medial and a lateral bony crest. Caudally, this surface continues with a reduced posterodorsal process, the coronoid process (*Processus coronoideus*), for articulation with the coronoid bone. The right and left lateral bony lines meet in a sagittal plane and form a sharp triangular bony projection. This bony projection continues caudally, with a midline crest flanked by two excavated areas. The lateral surface (*Facies lateralis*) decreases its height in a caudo-rostral direction and is perforated by multiple vascular foramina (*Foramina vascularis*) and nervous foramina (*Foramina nervosa*). Also, near the coronoid process, the foramen dentofaciale majus could be observed. Caudally, one posterolateral process, the surangular process (*Processus surangulare*), continues the lateral surface and connects to the surangular bone. The medial surface (*Facies medialis*) of the dentary bone bears a longitudinal excavation which forms the Meckel’s fossa (*Fossa meckelii*) and allows identification of the inferior alveolar foramen (*Foramen mandibulae*).

The coronoid bone (*Os coronoideum*) (Figure 6) is the smallest component of the lower jaw. Its dorsal coronoid eminence (*Eminentia coronoidea*) forms the highest point of the mandible. Posterior to the previously mentioned eminence, the coronoid bone bends postero-ventrally and articulates with the surangular bone laterally. Its medial surface sends a medial process (*Processus medialis*), which meets the prearticular bone medially and caudally.

The angular bone (*Os angulare*) (Figure 6) is an elongated lamellar structure that forms the postero-medial segment of the mandible and the caudal segment of the floor of the Meckel’s fossa. Its rostral process (*Processus rostralis*) lies over the medial aspect of the dentary bone, and its caudal process is flanked by the prearticular bone medially and the angular process of the dentary bone ventrolaterally. The caudal end of the posterior process of the angular bone meets the rostral segment of the articular bone (*Os articulare*).

The surangular bone (*Os surangulare*) (Figure 6) is a rectangular bony piece that forms the lateral aspect of the posterior 1/3 of the mandible. The lateral surface is smooth, and near its ventral edge bears the lateral adductor’s crest. Its second surface faces medially and forms the lateral wall of the caudal segment of Meckel’s fossa. Rostrally, this bone is higher and articulates with the coronoid bone rostro-dorsally and with the dentary bone rostro-ventrally. The posterior extremity is shorter and joints with the articular bone postero-dorsally and with the prearticular bone postero-ventrally. Ventrally, the surangular bone is bordered by the angular process of the dentary bone.

The prearticular bone (*Os prearticulare*) (Figure 6) is a lamellar bony structure opposite to the surangular bone and on top of the angular bone. Due to its position, it forms the medial part of the posterior 1/3 of the mandible. Its medial surface faces medially towards the intermandibular space, while its lateral surface forms the medial wall of the Meckel’s fossa. Dorso-rostrally, the prearticular bone is flattened laterally and reaches the medial process of the coronoid bone.

The articular bone (*Os articulare*) (Figure 6) is the most caudal bone of the lower jaw. It fills the space between the posterior segment of the surangular bone, laterally, and the posterior segments of the angular and prearticular bones, postero-medially, and forms the posterior end of the Meckelian fossa. Dorsally, the articular bone bears a slightly excavated articular surface (*Facies articularis*), which receives the mandibular condyle of the quadrate bone. The lateral part of the previously named articular surface is provided by the articular process of the surangular bone (*Processus articularis surangulare*). Rostrally, the articular surface is continued by a triangular rostral process, the angular process (*Processus angulare*), which enters into the Meckelian fossa and dorso-caudally by a bony projection, the retroarticular process (*Processus retroarticularis*).

### 3.2. Osteometrical Data

The following data sets were recorded on the available specimen (Table 2).

As the cranial measurements are important for the morphology, the taxonomical and functional studies, the quantification of data connected to the length of the cranium, the width of the cranium, and other measurements, such as the orbital dimensions, may contribute, in a much larger perspective, to the deeper understanding of some evolutionary facts, possibly adaptive changes, and developmental sequences.

The comparison and integration of such data enable a more robust framework, enhance the reliability of measurements and frames, and place local variation in a much larger context.

**Figure 7:**
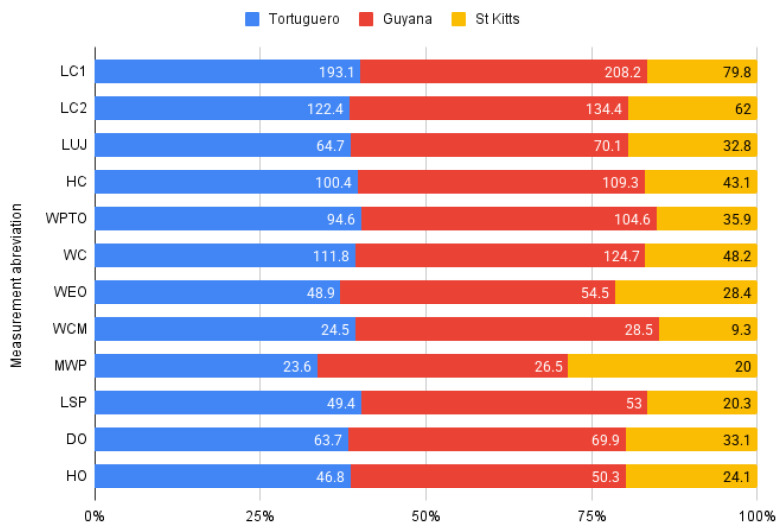
Comparative chart for cranial measurements. Average values for the indicated measurements (mm).

The standard use of the raw metrical data does not, in this case, provide much important information, as the collected data originate from a juvenile individual, showing only that the comparative measurements valid for adults place our studied specimen. Among the available data, we focused on geographic areas close to the one where our specimen originates, using metrical data from samples from the Tortuguero region and Guyana, both located in the Atlantic region [33]. As the comparative sample contains only mature individuals, we made some simple calculations to illustrate the ratios of adult vs. juvenile measurements, as displayed in the graph (Figure 7):

## 4. Discussion

From a metrical perspective [33], having in mind three of the main measurements dealing with the length of the overall cranium lengths (LC1 And LC2) and the overall width of the cranium (WC), we can infer the fact that our juvenile individual has reached about 40% development of an normal adult as seen in measurements of the Chelonians in the area of the Atlantic in absolute terms (Figure 7, Table 3).

The calculation of the most common indices (Table 3) (the length of the cranium and the width of the same element) suggests, in fact, some interesting facts. It is clearly visible that, despite the dimensional difference among juvenile and adult, the total length/width ratio remains constant in both types of individuals, maintaining a 1.6–1.7 value for the LC1, as the most anterior part seems to have a slightly higher value, when LC2 (that expresses the premaxilla-quadrate bone length) may indicate a slightly shorter segment in this stage.

To verify the previously mentioned aspect, we calculated another index that compares the length of the upper jaw (LUJ) to the length of the cranium. The calculation confirms a reduction in the length of the most anterior structures of the skull, including the maxillary bone length.

A similar approach attempted to calculate the influence of the cranium’s length relative to its overall height [34]. As we can observe, the values are similar for juveniles and adults for overall length, as the anterior part of the cranium still shows possible later development, appearing consistent with earlier conclusions [19].

Orbital dimensions also play a role in the expression of the skull’s facial characteristics [34]. Similarly, another calculated index attempts to assess the variation in these dimensions relative to the overall height of the skull. Our findings show very similar orbital index values for all investigated adult and juvenile specimens. In comparison with the overall height of the cranium, the proportions are not maintained, as the height of the skull in its posterior part (as reflected by the HO measurement) is different in juveniles compared to the adults investigated.

Data for the mandible were not computed due to a lack of comparative data.

The macroscopic investigation revealed that in a *Chelonia mydas* juvenile specimen, the skull shows elements similar to those reported in Green turtles [19,23,28,36], other turtles [9,16,23,27,30,37,38,39], lizards [30,40,41,42,43], and snakes [36].

As sources [23] describe, in our specimen, the upper jaw is U-shaped and forms the rostral segment of the splanchnocranium. This aspect differs from that reported for *Caretta caretta*, *Lepidochelys kempii*, *Lepidochelys olivacea*, *Eretmochelys imbricata* and *Dermochelys coriacea*, where the jaws have more or less pronounced V-shapes [16,23,30].

In our juvenile specimen, as reported in turtles [9,16,28,30,37] and other reptiles [30,36,38,39,40,41], the skull shows paired and unpaired bones.

The most rostral bone of the splanchnocranium was the paired premaxillary bone, which in our specimen formed the anterior tip of the snout. This bone has a similar disposition in other turtles, such as *Stylemys nebrascensis*, *Gopherus polyphemus*, *Manouria impressa* [37], *Caretta caretta* [9,30], *Lepidochelys kempii* [9], *Alligator mississippiensis* [42], and some lizards [30,39,41]. In our specimen, as in *Stylemys nebrascensis* and *Manouria impressa,* the palatine surface of the premaxillary bones is smooth. Different aspects were mentioned in Gopherus *polyphemus,* where the interpremaxillary suture forms a sharp premaxillary ridge that protrudes into the buccal cavity [37]. In *Varanus komodoensis* [42] and *Cyclura carinata* [40], the premaxillae are fused and form a single bony piece. Compared with the macroscopic features reported in turtles, lizards, and alligators, the ventral margin of the premaxillary bone has a tooth-bearing alveolar plate, allowing identification of a variable number of premaxillary teeth [30,39,40,41,42,43].

The maxillary bone is well developed, and our findings confirm the data reported by [28]. Similar information regarding the general disposition of the maxilla has been noted in other turtles, including *Caretta caretta*, *Lepidochelys kempii*, *Styloglossa nebrascensis*, and *Opherus polyphemus* [40]. In the studied specimen, the pterygo-maxillary suture was not identifiable.

The regional anatomy of the skull shows that, as reported in the literature [9,30], the maxillary bone forms a significant segment of the walls of the orbital cavity in *Chelonia mydas*. In *Iguana iguana* [30], *Ctenosaura pectinata* [39], *Echinosaura horrida* [41], *Cyclura carinata* [40] and *Alligator mississippiensis* [43], the maxillary bones articulate with the nasal bones. Specific for all the previous species of lizards and for *Varanus komodoensis* [42], the ventral margin of the maxillary bone bears dental alveoli for the maxillary teeth.

In the juvenile *Chelonia mydas* investigated, as in other turtles, the prefrontal bone is an important bony element forming the walls of two cranial cavities: the nasal and orbital cavities [30]. The presence of the lacrimal orifice, which perforates the nasal process of the prefrontal bone to continue with the nasolacrimal duct, reported by us in Green turtle, has been confirmed by [37] in *Stylemys nebrascensis*, *Gopherus polyphemus* and *Manouria impressa* and by [44] in *Proganochelys quenstedtii*. In *Varanus komodoensis*, the lacrimal foramen is margined by the ventral process of the prefrontal bone and the lacrimal bone [42]. The prefrontal bone is also part of the orbital cavity in *Alligator mississippiensis* and *Echinosaura horrida* [41,43].

As in *Caretta caretta* and *Lepidochelys kempii specimens*, the frontal bone in our specimen was paired and formed part of the skull roof. The V-shaped aspect of the fronto-prefrontal junction reported by us in our specimen is similar to the reported data in *Stylemys nebrascensis*, *Gopherus polyphemus*, *Caretta caretta*, and *Lepidochelys kempii*. However, in *Manouria impressa*, the same suture has been reported to have a U-shape [9,30,37]. The lateral junction of the frontal bone with the prefrontal and postorbital bones allows it to form a small segment of the dorsal orbital margin in the juvenile *Chelonia mydas* studied specimen. Although this feature is similar to that reported in *Lepidochelys kempii*, *Lepidochelys olivacea*, and *Eretmocelys imbricata* [9], in *Gopherus polyphemus,* due to the size and position of the parietal bone, the fronto-postorbital contact is absent [37]. In *Caretta caretta*, due to the presence of the prefrontal-postorbital contact, the frontal bone is not part of the orbital margin [9,30]. In iguanas, other lizards, and alligators, the frontal bone has been described as an important component of the brain case roof. Its ventral surface is smooth and forms a tubular structure that surrounds the olfactory tracts [39,42,43,45,46].

The postorbital bone in the investigated juvenile of *Chelonia mydas* shows the same contacts as those of the postorbital bones of *Manouria impressa*, *Gopherus agassizii*, and *Gopherus flavomarginatus* [37]. Important to note that, in *Echinosaura horrida*, due to the presence of the postfrontal bone, the postorbital bone is not part of the orbit [43]. According to [42], in *Varanus komodoens*, the fusion of the postorbital and postfrontal bones forms a complex structure (the postorbitofrontal bone).

The last component of the orbital margin in our specimen was the jugal bone. As reported, in contrast to our findings, the medial process of the jugal bone is absent in *Tortudo marginata*, *Gopherus polyphemus*, and *Manouria impressa*, and this is associated with the absence of the palato-jugalis and pterygo-jugalis sutures [37]. In *Caretta caretta* [30], the jugal bone is reported as L-shaped, whereas in Platysternon megacephalum [9] it is very small. A large jugal bone has been reported in the eastern mud turtle (*Kinosternon subrubrum*) [47]. In *Caretta caretta* and *Lepidochelys kempii*, the authors reported that the jugal bone occasionally showed a caudal spur [9]. In lizards, the jugal bone has a position similar to that reported by us in *Chelonia mydas* and by other authors in other turtles [9], but it also shows supplementary articulation with the lacrimal bone [39,40,42]. Refs. [9,47] reported the lack of lacrimal bone in all extant turtles. In adult specimens of *Cyclura carinata* (*Squamata: Iguanidae*) [40] and *Echinosaura horrida* (*Gymnophthalmidae: Cercosaurinae*) [41], the presence of a few lateral foramina has been reported near the ventral margin.

The anatomical disposition of the quadratojugal bone in *Chelonia mydas* was similar to the reported data for *Gopherus polyphemus*, with evident articulations with the jujugal rostrally, the postorbital rostro-dorsally, squamosal dorso-caudally, and the quadrate caudally. A small difference has been documented in *Manouria impressa*, where rostrally, only the postorbital bone articulates with the quadratojugal bone [37].

Reported as one of the bones of the skull in turtles [16,23,30] and birds [32,39,48], the quadratojugal bone has been reported as absent in iguanid lizards.

The anatomical contacts described in our specimens of the quadrate bone are similar to those reported for *Stylemys nebrascensis*, *Gopherus polyphemus*, and *Manouria impressa* [37]. The foramen stapedio-temporalis, reported by us in our specimen, has been reported by [49] in four other families of turtles (*Chelydridae*, *Cheloniidae*, *Emydidae* and *Testudinidae*).

In contrast to turtles, in which the quadrate has fixed articulations with the bones of the skull [49], in iguanid lizards, syndesmotic and synovial joints have been reported, permitting some movements [39]. Also, for lizards, the quadrate bone has been reported as having two different condyles: the mandibular condyle (part of the craniomandibular joint) and the cephalic condyle for squamosal and supratemporal bones [30,39,40,41,42].

The squamosal bone in *Caretta caretta* [9,30] shows the same contact points as those reported by us in the juvenile *Chelonia mydas* individual [37]. reported different aspects in *Stylemys nebrascensis*. In *Varanus komodoensis*, CT data reported the absence of direct bone-to-bone contact between the squamosal and quadrate bones [42].

A very important bone of the roof of the skull in our specimen and other turtles was the parietal bone. According to [9,30], in *Caretta caretta* and *Lepidochelys kempii*, these bones are the largest in the skull. In our specimen, its ventral surface bears a ventral process, indicated by us and [28] as the *ala major ossis sphenoidalis*, or by other authors as the *processus inferior parietalis* [30]. Due to the presence of this bony process, the ventral surface of the parietal bone will medially form the roof of the *cavum cranii* and the roof of the *fossa temporalis* laterally. The caudal margin of the temporal bone plays an important role in skull identification.

Similarly to our findings, ref. [23] reported shallow parietal notches for *Chelonia mydas*, but large parietal notches for *Caretta caretta*, deep parietal notches for *Lepidochelys olivacea* and *Eretmochelys imbricata*, and also reported the lack of parietal notches in *Dermochelys coriacea* turtles.

It has been reported as an unpaired bone in *Varanus komodoensis* [42], *Echinosaura horrida* [41], *Cyclura carinata* [40], and *Ctenosaura pectinata* [39]. According to [42], the parietal bone and the frontal bone build the main part of the skull in *Varanus komodoensis*.

The caudal end of the *cavum cranii* allows us to note that the occipital area has a few bony pieces. The supraoccipital and basioccipital bones, as unpaired elements, formed the caudo-dorsal and ventral and caudo-ventral segments, while the exoccipital bones, pair bones, will form the lateral segments. All four bones surround the occipital foramen (*Foramen magnum*). Similar findings have been reported in *Cyclura carinata* and *Ctenosaura pectinata* [39,40].

Because in some species the exoccipital bones fuse with the opisthotic bones to form the otooccipital bones, the borders of the foramen magnum can vary [30,40,41,42,50]. As in *Caretta caretta*, in our specimen, the supraoccipital bone extends caudally with an evident supraoccipital crest [9].

Similarly to the data reported by [37] in *Stylemys nebrascensis*, *Gopherus polyphemus*, and *Manouria impressa* and by [39], in *Ctenosaura pectinata* or in our specimen, the occipital condyle is a tripartite structure formed by fusion of the most caudal part of the basioccipital bone with the ventro-caudal part of the exoccipital bones. The hypoglossal canals we reported on the surface of the exoccipital bone are similar to those reported in *Stylemys nebrascensis* [37].

Different variations have been reported in *Cyclura carinata* [40], *Echinosaurhorrida* [41] and *Varanus komodoensis* [42], in which the authors describe hypoglossal foramina located close to the vagus foramen on the surface of the otooccipital bones.

Rostrally, the basisphenoid bone fills the space between the basioccipital and pterygoid bones. This feature confirms the literature-reported data in other reptiles [40,41,42,50].

The pterygoid bone has filled the space between the palatine and basisphenoid bones. Unlike the morphological aspects reported by us in *Chelonia mydas*, on the palatal surface of the pterygoid bones in Cyclura carinata, Amblyrhynchus, nd *Iguana*, pterygoid teeth have been reported [40,51,52]. The same structures are variable in desert iguanas (Dipsosaurus dorsalis) and are absent in Galápagos land iguanas (*Conolophus subcristatus*) [39,40].

The vomer, together with the palatine, maxillary, and premaxillary bones, form the wall between the nasal and buccal cavities. Similar contacts have been reported in *Lepidochelys kempii* (and other chelonioids) and in *Stylemys nebrascensis* [9,37]. According to [9,23], in Caretta caretta, the rostral contact between the vomer and premaxillary bones is absent.

The ventral excavation of the vomer reported by us on the triturator surface has also been described in *Gropherus* specimens [53]. Although in our specimen this bone is impaired, in *Cyclura carinata*, it was reported as a paired structure in contact with the ventro-posterior segment of the septomaxillary bone [40].

In turtles, the vomer, maxillary, and premaxillary bones of the upper jaw, and the dentary bones of the lower jaw, are covered by keratinous structures known as the upper and lower rhamphotheci [23].

The right and left mandibles are the only movable elements of the cephalic extremity. On each side, five bones could be observed: dentary, angular, surangular, coronoid, prearticular, and articular bones. Rostrally, the right and left mandibles have been fused at the level of the intermandibular symphysis. Our findings confirm the data reported by [9,30]. One supplementary bone, the splenial bone, has been reported in *Alligator mississippiensis* [43], *Varanus komodoensis* [42], *Ctenosaura pectinata* [39], *Echinosaura horrida* [41] and *Cyclura carinata* [40]. The presence of the intermandibular symphysis has been reported in *Cyclanorbinae* and *Trionychinae* [37,54].

The absence of the intermandibular symphysis and the existence of the splenial bone as part of the mandibles have also been reported in *Chelids* [54,55]. The presence of the medial and lateral ridges described by us on the dorsal surface of the dentary bones confirms the reported data for *Stylemys nebrascensis* and *Gopherus polyphemus*. They can also be named the labial and lingual ridges [37,54]. It is important to note that in other reptiles, the dorsal margin of the dentary bone bears a variable number of dental alveoli [38,39,40,41,42,43,50].

The morphologic features of the coronoid bone in our specimen were similar to those reported for *Stylemys nebrascensis*. A more reduced coronoid process has been illustrated in other testudinids, where it was almost as high as the highest point of the dentary [37,54].

As in other reptiles [41,42], in the investigated *Chelonia mydas* specimen, the caudal margin of the coronoid bone formed the rostral edge of the Meckel’s fossa. The angular, surangular, prearticular, and articular bones surround the caudal end of the Meckel’s fossa, and the articular bone, together with the dorso-caudal ends of the surangular bone laterally and the prearticular bone medially, form the cranio-mandibular joint.

## 5. Conclusions

Based on the raw metrical data available for our investigated specimen, we can state that the metrical data indicate an individual that has reached about 40% of its overall dimensional development. The rate of elongation during normal development of the skull seems to favour the posterior part, while indices involving the anterior part show only a small difference in proportions, suggesting a later elongation of the anterior part. The skull’s height undergoes similar processes. However, the overall height is slightly lower than this tendency in the orbital (anterior) part. This fact may also suggest a functional priority for neurocranial protection before the complete development of the masticatory apparatus or the possible changes imposed by feeding habits.

The integration of metrical and integration data, along with strong descriptive morphological data, provides a reference for taxonomic, functional, and possibly clinical studies, offering insights into relevant anatomical data for veterinary diagnostics or general biological studies.

## Figures and Tables

**Table 1:** List of measurements and used abbreviations for the skull measurements.

Measurement Abbreviation	Explanation
LC1	Cranial length from the tip of the premaxilla to the posterior supraoccipital
LC2	Cranial length from the tip of the premaxilla to the tip of the condylar process of quadrate bone
LUJ	Length of the upper jaw
HC	Height of the cranium—maximum distance between the most upper part of the posterior parietal bone and the ventral part of the upper jaw (maxillary bone)
WPTO	Width of the postorbital—maximum transversal distance between the postorbital
WC	Width of the cranium—maximum distance at the level of the mandibula condyle
WEO	Width of the exoccipital—maximum distance between the lateral projections of the bone
WCM	Width of the mandibular condyle
MWP	Minimum width across the pterygoid bone
LSP	Length of the secondary palate—sagittal length measured from the tip of the premaxilla to the posterior vomer border
DO	Maximum diameter of the orbit
HO	Height of the orbit
HN	Height of the nasal opening
WN	Width of the nasal opening
HPM	Height of the premaxilla (close to the sagittal margin)
MDON	Minimum distance between the orbit and nasal opening
WSO	Width of the supraorbital
WPO	Width of the preorbital
WZ	Width of the zygomatic
WSM	Width between the squamosals
HM	Height of the mandibula
LJA	Length of the mandibular symphysis
WM	Width of the mandible
LM	Length of the mandible from the anterior tip to the most posterior point

**Table 2:** Available skull measurements for the Green Turtle specimen.

Measurement	Value (cm)
LC1	7.98
LC2	6.20
LUJ	3.28
HC	4.31
WPTO	3.59
WC	4.82
WEO	2.84
WCM	0.93
MWP	2.0
LSP	2.03
DO	3.31
HO	2.48
HN	-
WN	-
HPM	-
MDON	0.34
WSO	2.30
WPO	2.17
WZ	4.18
WSM	4.51
HM	-
LJA	0.73
WM	3.57
LM	4.87

**Table 3:** Calculated cranial indices for the investigated Green Turtle specimen and comparative data from other geographic regions.

Cranian Indices	Tortuguero	Guyana	StKitts
Ratio LC1/WC	1.727	1.669	1.655
Ratio LC2/WC	1.094	1.094	1.286
Ratio LC1/LC2	1.577	1.549	1.287
Ratio LUJ/LC2	0.528	0.521	0.529
Ratio LUJ/LC1	0.335	0.336	0.411
Ratio HC/LC1	0.519	0.524	0.540
Ratio HC/LC2	0.820	0.813	0.695

## Data Availability

The data supporting the findings of this study are derived from the direct examination of museum cranial specimens of turtle housed in the institutional RUSVM’s Centre for Integrative Mammalian Research osteological collection. Specimen-level data (including catalogue numbers, morphometric measurements, and observational records) are available from the corresponding author upon reasonable request and with permission from the RUSVM’s Centre for Integrative Mammalian Research. Access to physical specimens is subject to the policies and regulations of the respective museum collection. No new specimens were collected for this study.
